# L’embolie pulmonaire au centre hospitalier universitaire Campus de Lomé (Togo): étude rétrospective à propos de 51 cas

**DOI:** 10.11604/pamj.2017.27.129.6855

**Published:** 2017-06-18

**Authors:** Soulemane Pessinaba, Yaovi Dodzi Molba Atti, Soodougoua Baragou, Machihude Pio, Yaovi Afassinou, Mohamed Kpélafia, Edem Goeh-Akué, Findibé Damorou

**Affiliations:** 1 Service de Cardiologie du CHU Campus, Lomé, Togo; 2 Service de Cardiologie du CHU Sylvanus Olympio, Lomé, Togo

**Keywords:** Embolie pulmonaire, épidémiologie, diagnostic, traitement, Togo, Pulmonary embolism, epidemiology, diagnosis, treatment, Togo

## Abstract

**Introduction:**

L’objectif était d’étudier les aspects épidémiologiques, cliniques et évolutifs de l’embolie pulmonaire au CHU Campus de Lomé.

**Méthodes:**

C’est une étude rétrospective, analytique et descriptive sur une période de 39 mois (1^er^Novembre 2011- 31 Janvier 2015). Etaient inclus, tous les dossiers des patients hospitalisés dans le service de cardiologie du CHU Campus pour une EP.

**Résultats:**

La prévalence de l’EP était de 3,1%. Le sex ratio femme/homme était de 2,2. L’âge moyen des patients était de 52,7 ± 14,4 ans. Les facteurs de risque de MTEV étaient dominés par: l’obésité (54,9%), l’alitement (25,5%) et le long voyage (17,6%). Les principaux symptômes étaient: dyspnée (98,0%), douleur thoracique (78,4%) et toux (60,8%). Le score de Wells était élevé dans 29,4%. L’ECG notait: tachycardie (78,4%), HVD (49,0%), aspect S1Q3T3 (47,1%) et bloc droit (39,2%). L’échodoppler cardiaque transthoracique montrait une dilatation cavitaire droite et thrombus intra ventriculaire droit dans 5,6%. L’angioscanner thoracique était normal dans 9,8% et objectivait un embole dans 82,4%. Le traitement était fait d’HBPM à dose curative avec relais par un AVK. Une thrombolyse était effectuée chez 8 malades. L’évolution était favorable dans 86,3%. Le taux de létalité était de 13,7%.

**Conclusion:**

La prévalence de l’EP est relativement faible chez nous mais probablement sous estimée. L’EP pose un problème thérapeutique au Togo à cause du coût élevé des examens complémentaires et de la thrombolyse. La prévention reste donc l’arme efficace.

## Abstract (en)

**Introduction:**

This study provides an analysis of the evolutionary, clinical and epidemiological aspects of pulmonary embolism at the University Hospital Campus of Lome.

**Methods:**

We conducted a retrospective, analytic and descriptive study over a period of 39 months (November 1 , 2011- January 31, 2015). All the medical records of patients hospitalized for PE in the Department of Cardiology at the University Hospital Campus were analyzed.

**Results:**

The prevalence of PE was 3.1%. Female/male sex ratio was 2.2. The average age was 52.7 ± 14.4 years. Risk factors for venous thromboembolic disease VTD were dominated by: obesity (54.9%), bedrest (25.5%) and long journey (17.6%). The main symptoms were: dyspnoea (98.0%), chest pain (78.4%) and cough (60.8%). Wells’ score was high in 29.4% of cases. ECG showed: tachycardia (78.4%), right ventricular hypertrophy (RVH)(49.0%), S1Q3T3 aspect (47.1%) and right block (39.2%). Transthoracic Doppler echocardiogram showed right cavitary dilation and right intraventricular thrombus in 5.6% of cases. Thoracic angioscanner was normal in 9.8% of cases and showed embolus in 82.4% of cases. Treatment was based on Low Molecular Weight Heparin (LMWH) at therapeutic doses with antivitamin K (AVK) relay. Thrombolysis was performed in 8 patients. Evolution was favorable in 86.3% of cases. Case-fatality rate was 13.7%.

**Conclusion:**

The prevalence of PE is relatively low in our area but it is probably underestimated. PE is a therapeutic problem in Togo because of the high cost of complementary examinations and thrombolysis. Prevention is therefore the only effective weapon.

## Introduction

L’embolie pulmonaire (EP) se définit comme l’oblitération brutale (totale ou partielle) du tronc de l’artère pulmonaire ou d’une de ses branches par un corps étranger circulant, le plus souvent fibrino-cruorique [1]. Elle est secondaire à une thrombose veineuse profonde (TVP) dans 90 % des cas [2]. Elle constitue une urgence diagnostique et thérapeutique. En Europe, la prévalence de l’EP est de 17 à 42,6% des malades hospitalisés et de 8 à 52% des vérifications nécropsiques [3, 4]. Dans la population française l’incidence des MVTE comme diagnostic principal atteint 85,5 pour 100 000 habitants dont 61,7% pour l’EP [5]. En Afrique, les données restent encore difficiles à obtenir et la prévalence est sous-estimée [6]. Au Togo la prévalence de la MVTE en milieu hospitalier est de 3,1 % [7] mais aucune étude ne s’est penchée uniquement sur l’EP. Ainsi, les objectifs de ce travail est de décrire les aspects épidémiologiques, cliniques et évolutifs de l’EP au CHU Campus de Lomé, un des trois centres de référence du Togo.

## Méthodes

Il s’agit d’une étude de cohorte rétrospective et descriptive sur une période de 39 mois (1^er^ novembre 2011 au 31 janvier 2015) réalisé dans le service de cardiologie du CHU Campus de Lomé. Etaient inclus dans l’étude, les dossiers des patients des deux sexes et de tout âge hospitalisés pour embolie pulmonaire et ayant réalisé un angioscanner thoracique. Les données ont été collectées à l´aide d´une fiche d´enquête par un médecin du service. Le diagnostic d’EP était porté sur un faisceau d’arguments: cliniques: association de toux, de douleur thoracique, d’hémoptysie, de dyspnée de collapsus cardiovasculaire sur un terrain à risque avec l’évaluation du score de Wells [**8**] en fonction duquel les examens paracliniques sont réalisés; paracliniques: D Dimères, électrocardiogramme, échodoppler cardiaque, échodoppler veineux des membres inférieurs, angioscanner thoracique. Nous avons également étudié le traitement et l’évolution intra hospitalière. Les données ont été saisies sur le logiciel Word et Excel 2007 et analysées sur le logiciel SPSS version 22. Les tests statistiques ont été réalisés et le seuil de 5% considéré comme significatif pour la comparaison des données.

**Aspects éthiques:** L’approbation du comité d’éthique de l´université de Lomé était obtenue en accord avec les réglementations nationales et locales.

## Résultats

**Figure 1:**
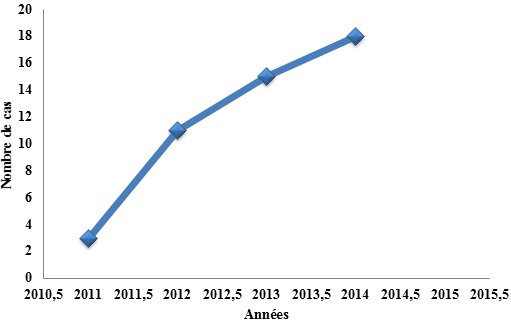
Nombre de cas d’embolie pulmonaire par année

La prévalence de l’EP était de 3,1% (51 cas/1622 patients hospitalisés). Il était noté une augmentation significative du nombre de cas d’EP diagnostiqués par année comme le montre la Figure 1. Sur les 51 patients, il y avait 35 femmes (68,6%) et 16 hommes (31,4%) avec un sex ratio F/H de 2,2. La moyenne d’âge était de 52,7 ±14, 4 ans avec des extrêmes de 27 et 81 ans. Les tranches d’âge de ]30-45 ans] et ]45-60ans] étaient les plus représentées. Cinquante trois pourcent des patients étaient admis dans le service par transfert ou référence, 27% venaient directement de leur domicile passant par le service d’urgence et seulement 20% étaient passés par la consultation cardiologique. Les symptômes présentés étaient: la dyspnée (98,0%), la douleur thoracique (78,4%), la toux (60,8%), l’hémoptysie (31,4%) et la syncope (29,4). La durée moyenne d’évolution des symptômes avant l’admission était de 5,6± 4,4 jours avec des extrêmes de 1 et 21 jours. Les facteurs étiologiques de MTEV retrouvés sont représentés dans le Tableau 1. L’hypertension artérielle et le diabète étaient présents dans respectivement 39,2% et 17,6% des cas.

**Tableau 1:** Facteurs étiologiques d’EP présents chez nos patients

Facteurs étiologiques	Nombre	Pourcentage
Obésité	28	54,9
Alitement prolongé	13	25,5
Long voyage	9	17,6
Accident vasculaire cérébral	7	13,7
Insuffisance cardiaque aiguë	6	11,8
Cœur pulmonaire chronique	3	5,9
Infection à VIH	3	5,9
Déficit en anti thrombine III	3	5,9
Drépanocytose	2	3,9
Post-partum	2	3,9
Chirurgie traumatologique	2	3,9

EP: embolie pulmonaire; VIH: virus de l’immunodéficience acquise

**Figure 2:**
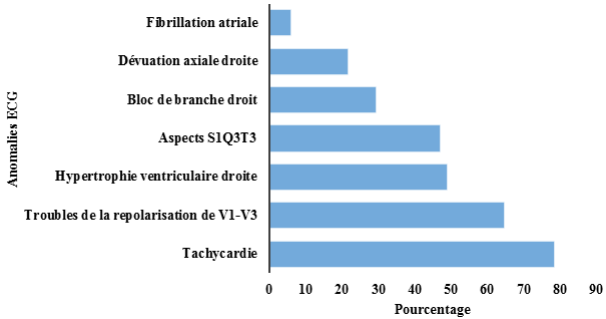
Signes électrocardiographiques retrouvés dans notre travail

Sur le plan clinique, 35,3% des patients avaient un état général altéré avec 21,6% en hyperthermie et 17,6% avaient présenté un collapsus cardiovasculaire. A l’examen cardiovasculaire on notait une tachycardie (80,4%), un éclat de B2 au foyer pulmonaire (60,8%), un souffle d’insuffisance tricuspidienne (51,0%), une arythmie cardiaque (15,7%), un bruit de galop droit (7,8%). Les signes d’insuffisance ventriculaire droite étaient objectivés dans 21,7% et seulement 5 patients (9,8%) avaient présenté des signes cliniques de thrombose veineuse profonde. Au niveau pleuro-pulmonaire, 47,1% avaient un examen normal, le syndrome de condensation pulmonaire et le syndrome d’épanchement pleural liquidien étaient notés dans respectivement 47,1% et 5,9%. Le score de Wells pour l’EP était faible dans 21,6%, modéré dans 29,4% et élevé dans 49%. La Figure 2 montre les signes électrocardiographiques de nos patients. A la radiographie du thorax 31,4% avaient une cardiomégalie, 21,6% des opacités alvéolo interstitielles et 9,8% une pleurésie. La dilatation de l’artère pulmonaire était retrouvée dans 5,9% l’ascension d’une hémicoupole diaphragmatique 3,9%.

Le dosage des D-dimères effectué chez les patients avec un score de Wells faible ou modéré (70,6%) était tous supérieur à 500 ng /ml par la méthode ELISA. L’étude des gaz du sang ainsi que le dosage des marqueurs biologique de souffrance myocardique (troponine I ou T) ou de dysfonction ventriculaire droite (BNP or NT-proBNP) n’étaient pas effectués. L’échodoppler cardiaque était réalisée chez 36 patients (70,6%) et les résultats sont présentés dans le Tableau 2. L’échodoppler veineux des membres inférieurs réalisé chez 7 malades retrouvait une TVP dans 4 cas.

**Tableau 2:** Anomalies retrouvés à l’échodoppler cardiaque transthoracique

Anomalies	Nombre	Pourcentage
Dilatation des cavités droites	27	75
Septum paradoxal	21	58,3
Hypertension artérielle pulmonaire	19	52,6
Dilatation du tronc de l’artère pulmonaire	17	47,2
Insuffisance tricuspidienne	15	41,7
Thrombus intra ventriculaire droit	2	5,6

**Figure 3:**
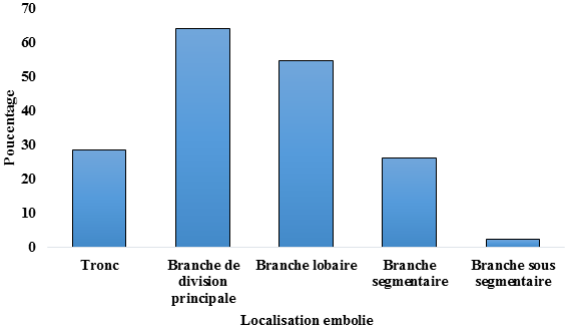
Localisation des emboles à l’angioscanner

A l’angioscanner thoracique, 9,8% avaient un examen normale avec un score de Wells modéré, un taux des D dimères élevé et un échodoppler cardiaque montrant un aspect de cœur pulmonaire aigu. Les emboles étaient objectivés chez 42 malades soit une sensibilité de 82,4%. Dans 57% l’EP était bilatérale, dans 26% elle était droite et dans 17% il s’agissait d’un EP gauche. La Figure 3 montre la localisation des emboles à l’angioscanner. Un infarctus pulmonaire était noté chez 58,7% des patients et une pleurésie chez 43,5%. Une thrombose de la veine cave supérieure était notée chez un malade.

Le traitement comportait dans tous les cas, de l’énoxaparine à dose curative initialement avec relais par les anti vitamines K (fluindione 80% et acénocoumarol 20%). Le traitement non spécifique était basé sur la mise au repos stricte les premiers jours d’hospitalisation, une oxygénothérapie, une administration d’antalgique au besoin, un remplissage vasculaire par des macromolécules et une perfusion d’amine vasopressive (dobutamine) chez patient ayant présenté un collapsus cardiovasculaire. Une fibrinolyse par streptokinase était effectuée chez 8 malades, réalisée entre les 96^ème^ et 120^ème^ heures d’hospitalisation. La durée moyenne d’hospitalisation était de 14,5± 5,9 jours avec des extrêmes de 3 et 29 jours et le coût moyen de la prise en charge variait entre 450000 et 600000 f CFA. Le taux de décès hospitalier était de 13,7% (7cas/51) et l’évolution immédiate était favorable dans 86,3% des cas. Les causes se décès étaient: choc cardiogénique (5 cas) et mort subite suite un effort de défécation (2 cas).

## Discussion

La prévalence hospitalière de l’EP dans notre étude était de 3,1%. En Afrique Subsaharienne les prévalences varient entre 1,4% et 7% en fonction des études [6, 9–12]. Par contre Dans les pays européens, la prévalence de l’EP varie entre 17- 42,6% des patients hospitalisés et 8-52% des vérifications autopsiques [3, 4] et dans la population générale l’incidence est de 100-200 pour 100000 habitants [13]. La prévalence de cette affection dans nos pays en voie de développement est probablement sous estimée à cause des difficultés d’accessibilité aux moyens de diagnostic.

Les patients de cette étude étaient relativement jeunes avec un âge moyen de 52,7 ans et environ 65% avaient moins de 60 ans. Ce même constat est fait par Diall dans son travail [9]. Ceci contraste avec les résultats des travaux en occident ou l’âge moyen est de 68 ans en Allemagne [14] et 67,6 ans en France avec une incidence croissante avec l’âge [5]. Plus de la moitié de nos patients étaient admis par transfert avec une durée moyenne d’évolution des symptômes de 5,6 jours. Ce retard d’admission agit sur la prise en charge et le pronostic de ces patients.

Les facteurs étiologiques et les signes observés sont classiques [2, 4, 9] mais il faut souligner la difficulté du diagnostic lorsque la symptomatologie est peu significative donc la nécessité d’une stratégie diagnostique et de prise en charge adéquate. L’évaluation du score de Wells à été un grand outil de cette démarche diagnostique dans la réalisation des différents examens complémentaires. Les tracés d’électrocardiogramme étaient très contributifs avec la mise en évidence des signes d’un cœur pulmonaire. La radiographie du thorax réalisée chez le tiers des patients était effectuée dans le cadre de la recherche de diagnostic différentiel d’autant plus que les signes sont peu spécifiques. Le dosage des DDimères était un élément essentiel de notre démarche diagnostique de par sa valeur prédictive négative forte. Effectué chez les patients avec un score de Wells faible ou modéré, il a permis la poursuite des investigations par la réalisation de l’angioscanner thoracique spiralé. Ceci est une attitude recommandée par des sociétés savantes [2, 13]. L’absence du dosage des BNP, NT-proBNP, troponines ne nous avait pas permis de faire correctement la stratification du risque de décès précoce lié à l’EP [2]. Mais en se basant sur le plan clinique, 17,6% présentant une hypotension artérielle ou collapsus cardiovasculaire étaient classé à haut risque.

Près de 71% des patients avaient effectué l’échodoppler cardiaque transthoracique et les trois quart présentaient des lésions faisant évoquer un cœur pulmonaire. Cet examen fourni assez d’éléments pour l’orientation diagnostique de cette pathologie. Cependant, dans une étude prospective publiée par Miniati, l’échocardiographie transthoracique était normale ou ne permettait pas l’identification de 50 % des EP prouvées à l’angiographie [15]. Concernant l’angioscanner thoracique spiralé, sur les 51 effectués, 5 étaient normaux et chez 42 malades, un embole était objectivé ceci donnant une sensibilité de 82,4%. Notre étude ne nous permet pas de nous prononcer sur la spécificité de cet examen néanmoins dans la littérature, La sensibilité et la spécificité de l’angioscanner spiralé thoracique varient respectivement de 70 à 90 % et de 90 à 96 % selon les études et le type d’angioscanner [16–18]. Et du fait que cet examen n’est pas invasif, il est devenu le gold standard dans le diagnostic de l’EP en détrônant l’angiographie pulmonaire qui est plutôt un examen très invasif [2, 13]. Un examen angioscanner thoracique spiralé normal est un critère fondamental pour exclure l’EP chez les patients présentant un score clinique de probabilité faible ou intermédiaire. Cependant, chez les patients avec un score élevé, un examen normal nécessite la réalisation ultérieure de l’angiographie pulmonaire ou d’une scintigraphie de ventilation pulmonaire [2, 13].

Sur le plan de la prise en charge, l’arsenal thérapeutique restait conventionnel avec les héparines de bas poids moléculaire à dose curative et en relais un AVK. Le traitement fibrinolytique à la streptokinase a été effectué chez 8 patients. Plusieurs études ont comparé les traitements thrombolytiques et leurs modalités d’administration. Leurs résultats ont été intégrés dans une méta analyse récente évaluant l’efficacité de trois régimes de thrombolyses : alteplase en perfusion continue, alteplase en bolus et streptokinase. Cette méta-analyse ne montre pas de différence d’efficacité entre les trois schémas thérapeutiques mais montre un taux de décès moins important chez les patients traités par l’alteplase en perfusion continue [19, 20].

L’évolution était marquée par un taux de décès hospitalier immédiat de 13,7% avec une durée moyenne d’hospitalisation de 14,5 jours. Par contre en France [5] la létalité hospitalière de l’EP était de 5,4% en diagnostic principal et pour la durée d’hospitalisation, elle était de 10,1 jours en diagnostic principal. Cette différence peut s’expliquer par le retard d’admission, le coût élevé de la prise en charge estimé entre 13 et 18 fois le SMIG au Togo rendant ainsi un traitement optimal.

## Conclusion

La prévalence de l’EP est relativement faible au Togo comme dans les autres pays d’Afrique subsaharienne. Cette prévalence est probablement sous estimée. Dans notre étude, une prédominance féminine est observée et la population victime est relativement jeune. Il existe une multitude de facteurs de risque ou de comorbidités dont les principales retrouvées étaient l’obésité, l’alitement, le voyage prolongé, l’insuffisance cardiaque. Plus de la moitié des patients sont admis tardivement dans le service après un séjour dans d’autres structures hospitalières. Les aspects cliniques sont variés expliquant les difficultés diagnostiques. L’avènement de l’angioscanner facilite sa confirmation diagnostique. Elle pose un problème de prise en charge dans notre pays à cause du coût élevé des examens complémentaires et du traitement anticoagulant. Le taux de décès hospitalier est élevé. La prévention reste l’arme efficace dans la stratégie de prise en charge.

### Etat des connaissances actuelles sur le sujet

- Pathologie grave avec des signes cliniques non spécifiques;
- Facteurs étiologiques classiques connus et traitement bien codifié.

### Contribution de notre étude à la connaissance

- Contributions sur l’épidémiologie de l’embolie pulmonaire en Afrique; car les données sont rares dans cette région du monde;
- Obésité et alitement prolonge sont les facteurs de risque de MTEV les plus fréquents;
- La confirmation des facteurs étiologiques comme la drépanocytose et le VIH qui sévissent en Afrique.

## Conflits d’intérêts

Les auteurs ne déclarent aucun conflit d'interêt.
